# The Use of Yeast in Biosensing

**DOI:** 10.3390/microorganisms10091772

**Published:** 2022-09-02

**Authors:** Sudip Dhakal, Ian Macreadie

**Affiliations:** School of Science, RMIT University, Bundoora, VIC 3083, Australia

**Keywords:** biosensor, yeast, yeast two-hybrid, yeast surface display, FRET, fluorescent proteins, yeast reporters

## Abstract

Yeast has been used as a model for several diseases as it is the simplest unicellular eukaryote, safe and easy to culture and harbors most of the fundamental processes that are present in almost all higher eukaryotes, including humans. From understanding the pathogenesis of disease to drug discovery studies, yeast has served as an important biosensor. It is not only due to the conservation of genetics, amenable modification of its genome and easily accessible analytical methods, but also some characteristic features such as its ability to survive with defective mitochondria, making it a highly flexible microbe for designing whole-cell biosensing systems. The aim of this review is to report on how yeasts have been utilized as biosensors, reporting on responses to various stimuli.

## 1. Introduction

Many laboratory species of yeast are generally regarded as safe (GRAS) by the US Food and Drug Administration (FDA) and have been at the forefront of many advances in genomics, transcriptomics, and metabolomics. The budding yeast *Saccharomyces cerevisiae* has been the main workhorse in modern-day biotechnology [1]. It was the first eukaryote to have its genome sequenced in its entirety in 1996 [2]. Along with that advance was the concerted effort to assign functions to all 6000 open reading frames. The long history of yeast genetics provided an impetus for this work, which was followed by the systematic disruption of all open reading frames followed by an analysis of the ensuing changes [3]. The extensive genome duplication in *Saccharomyces cerevisiae* meant that in some cases strains needed to be made with multiple deletions to see a phenotypic change [4]. Nowadays, most of genes have functions assigned to them, and libraries of gene deletant strains are readily available.

The Gene chip technology was quickly developed in yeast and proved to be another convenient way to examine cellular responses after various stimuli. Some of the stimuli that have been examined were responses to various drugs, including prescription drugs for human diseases [5], responses to temperature, and responses to other stressors [6,7]. By synchronizing the growth of populations in culture, it also became possible to examine gene expression throughout the cell cycle [8]. Generally, this technology was easy to use but moderately expensive, and required the meticulous preparation of yeast mRNA. Therefore, there have been efforts to develop systems to enable reporting of responses in living cells using fluorescent reporters [9,10,11]. The development of reporter systems coupled with sensitive single-cell analyzers has enabled highly useful outputs. We describe some of the older and more recent advances as well as their applications in this review.

## 2. Yeast Biosensors in Protein–Protein Interactions

It is paramount to understand protein functions inside cells and how they interact with other biomolecules, especially in circumstances when the protein of interest is involved in disease pathogenesis or prevention. Protein interactions are quite common in biological systems and often serve in signaling pathways and the assembly of multi-sub-unit macromolecular complexes [12]. To date, several in vitro and in silico methods to detect protein–protein interactions have been devised; however, the interaction within an in vivo system is expected to be drastically different due to the presence of numerous biomolecules and spontaneously changing cellular microenvironment [13]. To address the in vivo interactions of the proteins involved, yeast biosensors have played a crucial role.

### 2.1. Yeast Two-Hybrid (Y2H) System

The Y2H system is one of the most powerful techniques for identifying protein–protein interactions in vivo. The Y2H technology stems from when Ptashne and colleagues characterized the Gal4p protein, which activates transcription in the presence of galactose [14]. The two domains of Gal4p, the N-terminal domain, and the C-terminal domain, can be expressed as separate domains. If the separated domains are able to interact and link covalently, the function of the Gal4p protein is reconstituted. 

The N-terminal fragment contains the DNA binding domain (DBD) and can bind the upstream activation site (UAS) but cannot activate transcription, while the C-terminal fragment contains the activation domain (AD) and is crucial for activating transcription of the DNA sequence downstream of the UAS [15,16,17]. An extremely innovative application of this knowledge led to the development of Y2H system in 1989 by Fields and Song [18]. The development of the Y2H system involved the simultaneous expression of Gal4p N-terminal fragment fused with a protein of interest (also referred to as bait) and the Gal4p C-terminal fragment fused with another protein of interest, referred to as prey. If the bait and prey proteins interact, Gal4p function is restored, due to the interactions of these two hybrid proteins. To conveniently determine the promoter activation, a reporter gene was inserted downstream of the UAS. 

**Figure 1:**
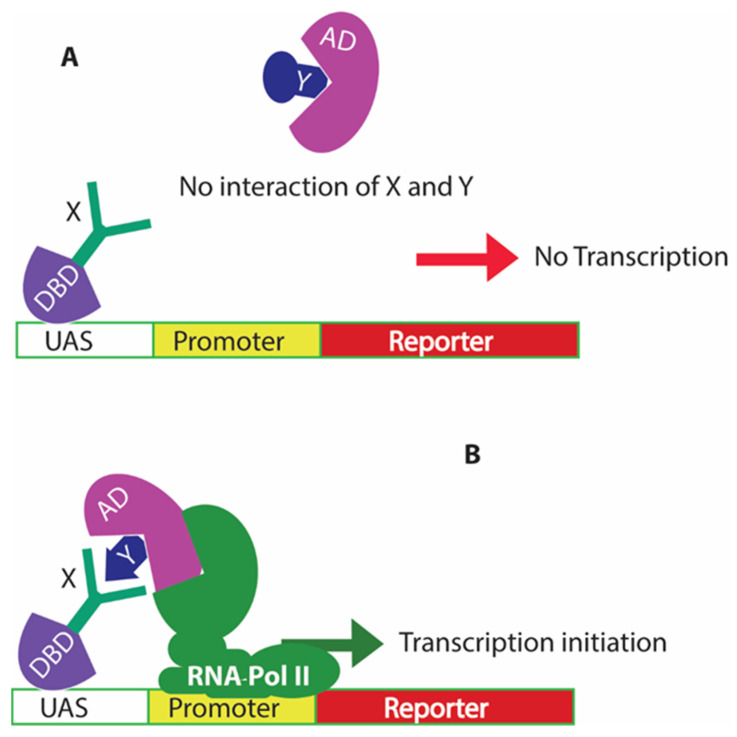
The classical yeast two-hybrid system for biosensing protein interactions in vivo. (**A**) shows there is no transcription of the reporter gene if there is no interaction between the proteins of interest X and Y; (**B**) shows that when there is an interaction of proteins X and Y, this leads to the recruitment of RNA polymerase II to the promoter region and activates the promoter for expression of the downstream reporter gene.

To validate the system, the researchers used SNF1-Gal4DBD fusion as the bait, SNF4-Gal4AD fusion as prey, and the *Gal1-lacZ* gene as the reporter (Figure 1). The reporter *Gal1-lacZ* gene encoded the beta-galactosidase enzyme and was used for evaluation of its expression using a colorimetric technique based on enzyme function. The study was crucial in showing the interaction between the two proteins and the usage of the method to evaluate the interaction inside a cell. 

Not only is the Y2H technology suited for determining whether proteins X and Y interact, but the technique can also be expanded to screen a library of prey proteins to search for one that interacts with a target protein bait [19]. Furthermore, and most importantly, the interactions are not in any way yeast focused. They can be used to screen for interactions between any proteins and are not limited to yeast-specific interactions within the cytoplasm of cells. Thus, many two-hybrid libraries have been constructed to discover novel protein interactions, including viral and host proteins [20,21]. Libraries that have been made commercially available include several tissue-specific cDNA libraries, including those from the human brain, heart, and lymphocytes [22,23,24]. 

Despite the benefits of using the classical Y2H system for identifying protein–protein interactions, there are also limitations, as assays can result in both false negatives and false positives [19]. Limitations such as the post-translational modification differences in yeasts and other eukaryotes, fusion proteins used in the study may not function as the intact native proteins, very short interactions time between the proteins, and protein interactions involving membrane proteins could result in false negative results [19]. At the same time, protein expression can be induced in yeast using inducible constructs and high copy plasmids can result in excessive amounts of proteins resulting in forced interactions inside the cell. Similarly, interactions of amyloid proteins can easily mislead the study, where interactions can result from the physical properties of the protein and not a specific interaction [19]. Various modifications of the classical Y2H system have been studied to elucidate the underlying interactions of the proteins (reviewed in [19,25,26]). Meanwhile, outcomes of the two hybrid analyses should be further evaluated by independent methods such as cross-linking experiments using materials from the original host. This may allow more reliable predictions about protein interactions identified using Y2H-based analysis. 

Following the discovery of the classical yeast two-hybrid system, a reverse two-hybrid system was also designed based on the same principles as the yeast two-hybrid system. The reverse two-hybrid system is also based on the expression of a reporter gene, but the reporter expression was designed to be detrimental to the cell, meaning that if the Gal4p function is reconstituted, then it results in cell death [27]. This system has allowed the identification of amino acids in the protein sequences that play critical roles in the protein–protein interactions [27,28]. For example, the reverse double two-hybrid system was used to identify missense mutations that disrupt the protein–protein interactions [28]. The system was referred to as a reverse double two-hybrid system as it combined both the reverse two-hybrid system as well as the classical two-hybrid system in one experiment. A triple fusion protein was generated, including the Gal4AD at the N-terminus, target protein mutant species in the middle (prey protein), and PTAP motif triple repeat units at the C-terminus. The Gal4DBD was fused with the bait protein or the protein of interest to which a mutation library was tested. If the truncated protein mutant is responsible for the disruption of protein–protein interaction, then the cells will not grow without uracil in the culture media and can be selected using 5-fluoro-orotic acid (5-FOA); however, if the protein interaction is not disrupted due to the mutation, then the cells will not grow with 5-FOA selection. On the other hand, the second interaction between the PTAP from the triple fusion protein with Tsg101 from the LexA-Tsg101 fusion was required to activate the lexA_op_ controlled expression of *HIS3*, which is essential for growing the cells in media deficit in histidine. This double selection was required to confirm that the effect in the protein interaction was due to a particular mutant protein species [28]. Apart from the above example, drugs that can interfere with known protein–protein interactions were also readily identified using the reverse two hybrid system signifying its importance in drug discovery studies [29]. 

### 2.2. Yeast Surface Display

The availability of eukaryotic machinery for post-translational modifications and ease of manipulating the genome has provided additional advantages for applying yeast for the surface display of proteins of interest [30]. Human proteins, including antibodies, cell surface receptor proteins, and protein targets of interest, such as cytokines, have already been displayed on the yeast cell’s surface [31]. The emergence of newer technological platforms for high throughput analysis in yeast has made the assays more favorable for yeast applications. 

**Figure 2:**
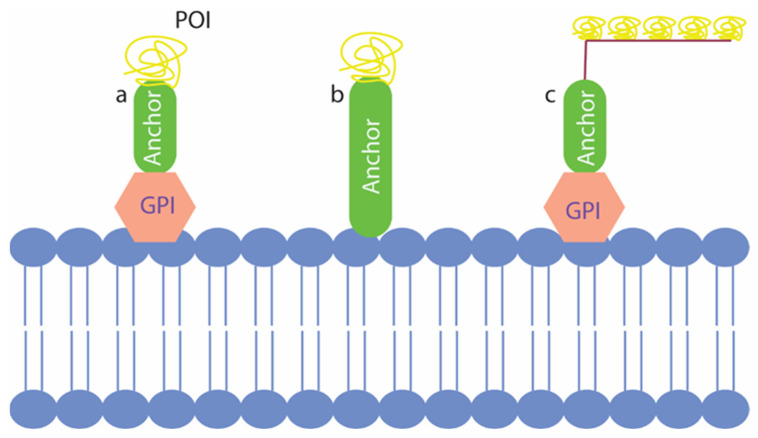
Three types of yeast surface displays that have been used in the past. (**a**) represents the classical yeast surface display with glycosylphosphatidylinositol (GPI) dependent anchor fused with the protein of interest (POI); (**b**) shows GPI independent anchor fusion with POI displayed in the cell surface; (**c**) shows co-display of multiple POIs on the cell surface using the GPI dependent strategy.

With the yeast surface display, the target protein or peptide of interest can be displayed on the cell surface while being expressed as a fusion protein with cell wall anchor proteins (Figure 2). For most yeast surface display systems, the anchor proteins are cell wall proteins linked with the glycosylphosphatidylinositol (GPI) [30]; however, there are GPI-independent anchor proteins such as Pir family proteins, which have been used to anchor some surface displayed proteins [32]. In general, a signal peptide, which directs the expressed protein into the ER lumen for post-translational modifications and required glycosylation of the cell wall anchor protein is crucial [33]. The expression system that expresses the fusion protein for yeast surface display can either be incorporated into plasmid vectors or can be integrated into the yeast genome. The proteins of interest can be expressed under the control of strong inducible or constitutive promoters to have optimal surface display [30]. 

Yeast surface display is another important example of yeast being used as a biosensor. Most of the applications of the yeast surface displayed proteins involve the study of protein–protein interactions [34]. In the past, these biosensors have been used in a variety of tasks, including biocatalysis, protein immobilization, protein engineering, vaccine discovery and production, antibody production and engineering, production of biofuel, and whole cell proteomic studies [34,35,36,37,38,39]. Recent advances involved the usage of the yeast surface display in detecting human disease, specifically COVID-19, by expressing antibodies and ACE2 receptor targeting spike proteins of SARS-CoV-2 on the yeast surface [40]. In another study, the SARS-CoV-2 spike receptor binding domain (S RBD) was surface displayed to identify escape mutants for the neutralizing antibodies [41]. In this way, the yeast-based biosensors have been useful in accelerating novel strategies against such highly contagious diseases. The protein interaction studies using yeast surface displays are not only limited to studying the interaction between two proteins of interest but have also been used to discover drugs. In a recent study, nanobodies against three distinct types of proteins of interest, including two membrane proteins, were selected from an immune library using the yeast surface display platform [42]. Thus, yeast surface display has served in multiple fronts, including its use in biosensing, and will prove to be instrumental in developing newer biosensors in the future.

## 3. Yeast Reporters

Numerous yeast biosensor reporters have been designed and applied in scientific studies to investigate important cellular mechanisms involved in diseases [43,44]. The conservation of proteins from yeasts to humans involved in fundamental cellular pathways makes yeast relevant in disease modeling [45]. Yeast reporters are mostly dependent on reporter gene expression under the control of inducible promoters [43]. These inducible promoters are activated during certain conditions by specific transcription factors. For example, a yeast reporter expressing mCherry red fluorescent protein under the control of a heat shock promoter containing heat shock elements has been designed to observe activation of the heat shock promoter [46]. Once the cellular conditions favor the HSF1 nuclear translocation, reporter activation is observed. This simple yet powerful tool can be used to screen for drugs that can activate the heat shock protein expression inside cells. As such, these heat shock proteins are crucial in important cellular protein quality control maintenance, which, when impaired, can lead to disease conditions [47]. Reporter systems in yeast biosensors can be based on fluorescence, luminescence, enzymatic reactions (usually measured with a color change in substrates), electrical signal, and growth rate [44]. Interestingly, yeast biosensors can be used to study the status of mitochondrial health, which is unique to yeast and cannot be performed in any other system [48]. Hence, yeast can be used as a biosensor to find chemicals that can modify mitochondrial health. A recent example of such a study found tyramine to impair respiratory growth in yeast cells in the presence of amyloid beta (Aβ) [49]. Impairment of respiratory growth restricts yeast growth on a non-fermentable carbon source such as ethanol. So, if ethanol is used as a sole source of carbon, tyramine causes a growth deficit, while the tyramine at the same concentration does not cause growth inhibition in media with glucose as the sole source of carbon. This property of yeast has been exploited and applied in identifying compounds that can modify mitochondrial health.

### 3.1. Biochemical Reporters

**Figure 3:**
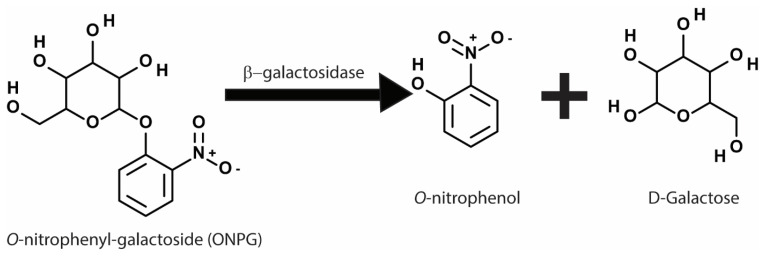
Chemical reaction involved in the β-galactosidase assay showing conversion of *o*-nitrophenyl-galactoside (ONPG) to *o*-nitrophenol and galactose. The *o*-nitrophenol produced can be quantified using colorimetry to measure absorbance at 420 nm wavelength.

The reporter constructs in the early phases of biosensor discovery utilized biochemical reporter genes. The *lacZ* gene from *Escherichia coli* which encodes the enzyme β-galactosidase, has been extensively used in all types of biosensor designs [50]. The β-galactosidase enzyme catalyzes a reaction that converts a colorimetric substrate *o*-nitrophenyl-galactoside to *o*-nitrophenol and galactose. The *o*-nitrophenol thus produced can be readily measured by light absorbance at 420 nm wavelength (Figure 3) [51]. The reporter is constructed in a way that the β-galactosidase expression is controlled by the promoter of interest becoming activated/induced upon receiving the stimulus of interest. If the assay is developed within the yeast two-hybrid system, the promoter activation requires the interaction of the two hybrid fusion proteins of Gal4p fragments [18]. Meanwhile, if the assay is directed to detect the expression of a heat shock promoter, a heat shock promoter will control the expression of the *lacZ* gene [52]. 

Despite the widespread use of *lacZ* to report various cellular events and protein–protein interactions, the expression of β-galactosidase and its functional assessment requires some laborious work as compared to newly designed fluorescence-based reporters: the assessment of β-galactosidase activity must be performed in a specific condition that allows the enzyme to be functional. For instance, β-galactosidase activity can be affected by a change in pH, which can change the output and the subsequent interpretation [53]. Meanwhile, fluorescence can be detected more accurately and rapidly in a high throughput manner. Fluorescent proteins that can resist pH fluctuations could be especially useful in conditions where pH change is inevitable [54]. Reporters comprising fluorescent proteins do not require extraction of proteins for measurement of activity: instead, they can be visualized inside cells under fluorescence microscopy or analyzed readily by flow cytometers [55].

Apart from the *lacZ* gene as a reporter, several genes encoding essential proteins required for particular auxotrophic yeast strains are also used as reporter genes. *HIS3*, *ADE2*, *URA3*, *LEU2*, and *TRP1* are some of the genes that can be used as the reporter gene depending on the strain of yeast under study [56]. 

### 3.2. Fluorescent Reporters

There are numerous fluorescent probes available, and many biotech companies have made probes suited for numerous applications. For example, DAPI, a very common DNA dye, can be used to stain the nucleus of cells as well as mitochondrial DNA and is particularly useful in microscopy [57]. In population analyses, DAPI staining can report on the ploidy of the population, giving indications about effects on the cell cycle [58,59].

There are many fluorescent chemicals that can be coupled to antibodies too, providing information on the structures to which those antibodies bind. This can be useful for visualizing sub-cellular structures and their cellular locations by confocal fluorescence microscopy [60]. In the case of fluorescence reporters, the best are fluorescent proteins, which have usually been first identified in nature, for example, those from fluorescent jellyfish [61]. Nowadays, there are a variety of such reporters whose genes have been cloned, sometimes modified further, and made available for exploitation. A summary of some of the useful fluorescent reporter proteins and their properties is shown in Table 1.

**Figure 4:**
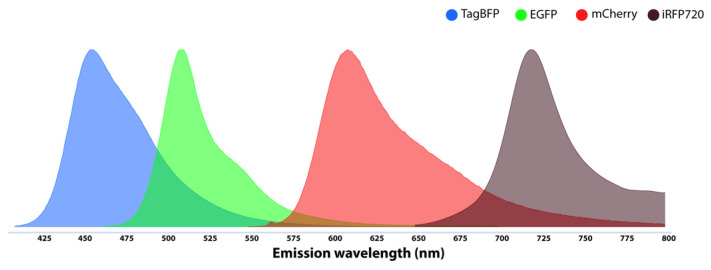
Emission ranges of some fluorescent proteins (TagBFP, EGFP, mCherry, and iRFP720) with fewer overlapping regions for designing a multi-colored biosensor in yeast.

So great is the number of fluorescent proteins that a database has been developed, providing a detailed list of fluorescent proteins that have been used in various studies as well as those that are not yet applied in yeast studies [62]. The beauty in their application is that the sequences encoding these fluorescent proteins can be fused to genes encoding proteins of interest to determine where those proteins of interest are located and how they are expressed inside a cell. In addition, sequences encoding fluorescent proteins can be placed downstream of promoters of interest so that one can gather information on the expression of those promoters in live cells [55]. To interrogate the yeast about more events at the same time, one needs to introduce more designs to overcome the challenges of co-expression of many fluorescent fusion proteins and/or fluorescent markers. The emission ranges of the different fluorescent proteins used in the reporter system should preferably not overlap substantially. Ideally, if possible, no overlap in the emission range of the two or more proteins should be considered. Hence, the selection of the fluorescent proteins must be made with caution, considering the final goal of the research (Figure 4). If there is a significant overlap in emission spectra, one needs to apply complex algorithms to compensate [63].

#### 3.2.1. Fluorescent Biosensors for Studying Neurodegenerative Diseases

**Figure 5:**
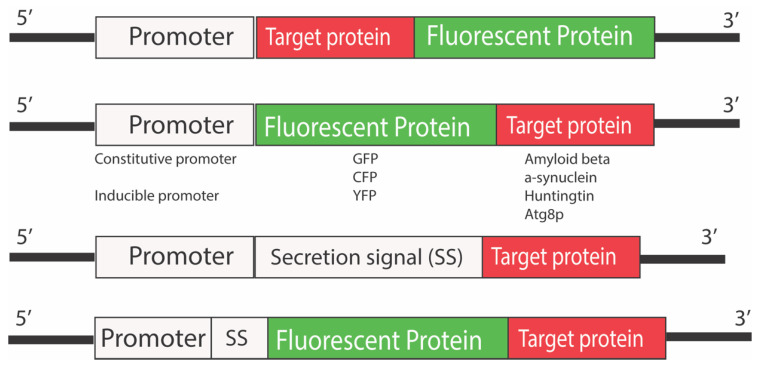
Yeast reporter constructs used in the study of neurodegenerative diseases. Different fluorescent proteins have been tagged at both N- and C-termini of the proteins of interest. The effect of tagging the target proteins of interest could be different depending upon the species of fusion protein. The promoters can be both constitutive or inducible based on the final application or goal of the study. A secretion signal can precede the fusion protein sequences if the ER processing of the fusion protein is required.

The expression of fluorescent protein tagged with disease-associated proteins is nowadays a common strategy for designing biosensors for studying diseases [64]. Proteins such as huntingtin, alpha-synuclein, and Aβ that are involved in Huntington’s disease, Parkinson’s disease, and Alzheimer’s disease, respectively, have been tagged with fluorescent proteins to study their role and to screen drugs [65]. Most commonly, green fluorescent proteins have been used as fluorescent markers, mostly due to the availability of several analytical platforms to detect the protein; however, other proteins, such as cyan fluorescent protein (CFP) and yellow fluorescent protein (YFP), have also been used to tag the proteins of interest (Figure 5).

Such yeast biosensors have provided enormous information on disease pathogenesis and ways to prevent the diseases, which was otherwise impossible. For example, in some pioneering work on Alzheimer’s Disease (AD) in our own studies, we examined the effects of Aβ [52]. Aβ is the protein that has been most associated with AD, and our studies have involved the expression of Aβ as well as GFP fused to Aβ, to examine its effects on yeast. 

Aβ probably has a somewhat non-specific effect on cells, with the deleterious effects being due to its accumulation. By studying yeast with GFP fused to Aβ we observed that in the population, despite all cells producing the fusion protein, young cells remove the fusion protein, so no young cells are fluorescent [48]; however, old cells retained the protein and had green fluorescence. This phenomenon is likely to be due to reduced proteostasis, a phenomenon that is also a significant part of human aging. It is highly likely that accumulated Aβ in older brain cells could lead to deleterious effects, including the death of neurons [66,67]. The yeast cells expressing GFP tagged Aβ have also been used for screening compounds and drugs that can act against such intracellular accumulation. Some studies have shown that simvastatin, latrepirdine, baicalein, and *trans*-chalcone reduce levels of Aβ in yeast cells [68,69,70]. Additionally, biosensors have also been applied to study the effects of combinations of compounds on intracellular Aβ [68]. A recent study identified the synergistic ability of baicalein and *trans*-chalcone combination to act against the Aβ and could be an effective way to treat or prevent the disease. In this way, yeast may provide information or aid in the generation of innovative ideas about the effects of Aβ on brain cells.

Apart from tagging the proteins directly involved in disease pathogenesis, yeast biosensors are also designed to report on important cellular mechanisms involved in cellular defense systems such as autophagy [70]. For example, to monitor the autophagic flux or drug-induced activation of autophagy, Atg8, a protein sequestered in the autophagosomal membrane when autophagy is activated, has been tagged with GFP. The fluorescence detection of the fusion protein has been used as the indicator for the autophagy activation signal. Latrepirdine is one example of such a drug identified as an autophagy inducer following the usage of such a yeast biosensor [70]. 

#### 3.2.2. Fluorescence-Based Heat Shock Response Yeast Sensor

**Figure 6:**
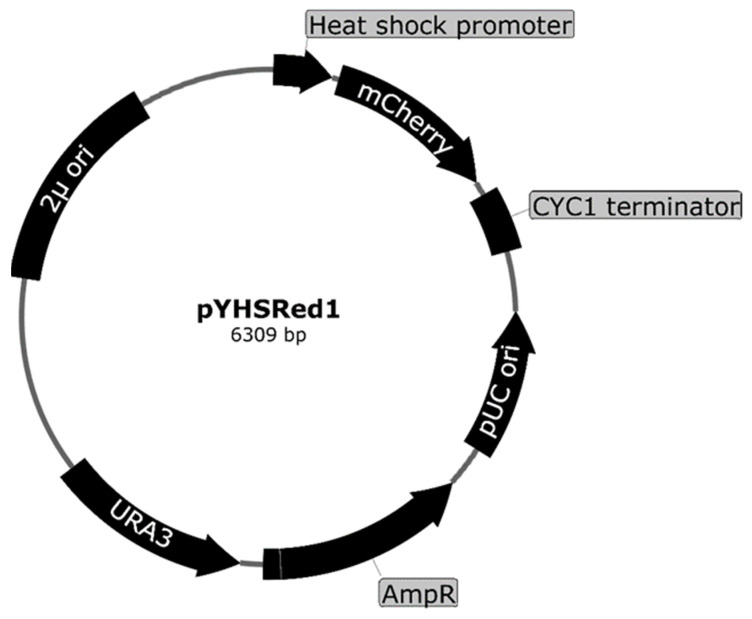
Plasmid map for the heat shock response reporter pYHSRed1 designed to express mCherry fluorescent protein as a reporter protein that expresses under control of a heat shock promoter, a promoter from heat shock protein 42 (*HSP42*) containing the heat shock elements (HSEs).

Genome-wide expression analyses in yeast producing the fusion protein showed that Aβ produced a stress response, which in yeast is referred to as the heat shock stress response (HSR) [71]. Heat can induce this response, but so can other factors, such as protein misfolding and reactive oxygen species (ROS) [72]. The heat shock response to Aβ was initially observed in genome-wide expression analyses and confirmed by introducing an HSR reporter plasmid into yeast to report on *lacZ* expression [52]. The HSR reporter yeast is a typical transcription factor (TF)-based biosensor as the biosensor design involves the expression of a reporter gene under the control of a heat shock promoter. The heat shock promoter in the biosensor contained heat shock elements (HSEs), which are recognized by the transcription factor heat shock factor 1 (HSF1) [73]. The compounds/ligands/biomolecules that can activate cytoplasmic HSF1 and aid its nuclear translocation will result in the expression of the reporter gene. Most recently, the HSR to Aβ was demonstrated in living yeast cells with a reporter plasmid expressing the fluorescent protein mCherry under the control of the HSR promoter when exposed to Aβ [55]. In the plasmid, the expression of the reporter gene encoding mCherry red fluorescent protein is controlled by the heat shock promoter (Figure 6). The mCherry fluorescent protein was incorporated into the design based on the protein’s ability to fluoresce in a wide range of pH, meaning that its fluorescence is not quenched in low pH organelles such as vacuoles/lysosomes [74]; however, similar fluorescent proteins can also be used in place of the mCherry fluorescent protein, and is subject to the future applications. In the assay, the activation of heat shock promoter by Aβ increased expression of the mCherry was analyzed using fluorescence microscopy and flow cytometry.

A host yeast strain that has multiple auxotrophic requirements is exploited for the selection and maintenance of transformants with a plasmids expressing Aβ as well as the heat shock response reporter. Each plasmid has a different selectable marker, enabling the selection and maintenance of both plasmids [52,63,69]. While both reporters just mentioned provide support that Aβ induces a stress response, the *lacZ* reporter is used in a biochemical assay of a population and is not very amenable to single cell analysis [52]. In contrast, the mCherry-based reporter is highly suited for single cell as well as population analyses, which includes studies involving fluorescence microscopy and flow cytometry. Thus, one can rapidly and conveniently investigate stress versus aging versus levels of Aβ fused to GFP. In the single cells of the population, information about cell size (related to age), green fluorescence (related to Aβ levels), and red fluorescence (related to cell stress) can be easily estimated. 

#### 3.2.3. FRET Microscopy-Based Biosensors

**Figure 7:**
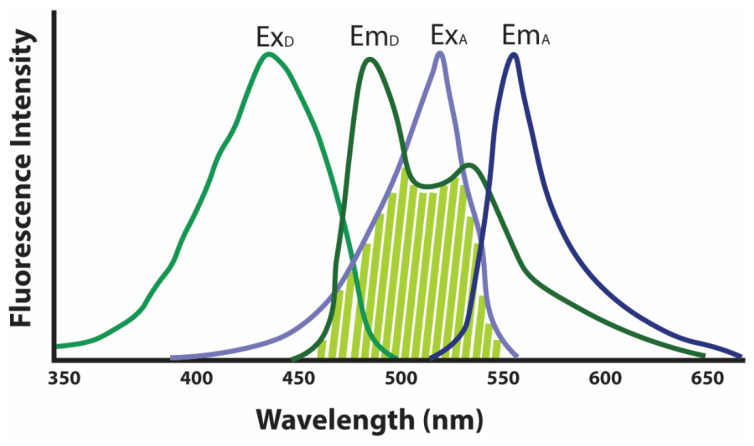
Excitation–Emission of the FRET donor (green curves) and FRET acceptor (blue curves) showing substantial overlap (shaded light green) in the range between FRET donor emission and FRET acceptor excitation. ExD, FRET donor excitation range; EmD, FRET donor emission range; ExA, FRET acceptor excitation range; and EmA, FRET acceptor emission range.

Forster resonance energy transfer (FRET) microscopy in yeast is another powerful fluorescence microscopy technique that has provided crucial information on the nanoscale assemblies of proteins in vivo [10]. FRET is a phenomenon of transfer of energy from a light-excited fluorophore (FRET donor) to another fluorophore (FRET acceptor) by dipole–dipole interaction when the two probes are present in close vicinity, where the distance between the probes should be below 10 nm [10]. Such energy transfer leads to the decrease in fluorescence emission from the FRET donor, while the fluorescence emitted by the FRET acceptor will increase as it obtains extra excitation energy during the transfer [75]. By analyzing the fluorescence shift, the interaction between the two fluorescence probes and the associated proteins or chemicals can be determined. The ease of expression of the fluorescent protein-tagged protein of interest in yeast makes it a convenient and attractive model for performing FRET analysis; however, the selection of specific fluorescent proteins as FRET donor and FRET acceptor is crucial for the successful experimental design [76]. A substantial overlapping region between the FRET donor’s emission range and the FRET acceptor’s excitation range is required (Figure 7). Other properties of the fluorescent proteins, such as photostability, the brightness of the proteins in vivo, and the distance between the molecules interacting, are also crucial in determining the FRET-based proximity [10,77]. It is possible for these FRET sensors to be analyzed using flow cytometry, which can give specific information on the underlying interactions.

Several FRET-based protein proximity studies have been performed in yeast species *S. cerevisiae* and *Schizosaccharomyces pombe.* Usually, in yeast, such biosensors are involved in the mapping of the organization of protein complexes and understanding molecular events in vivo. This technique has been successfully applied in understanding the organization of nuclear pore complex [78], cell division contractile ring [79], the nanoscale architecture of endocytic coat [80], and spindle pole body [81]. In such studies, pairwise proximities are determined, and the information obtained is analyzed to identify the protein map in the nano-environment. Apart from these abovementioned studies involving large protein complexes, several studies involving the study of smaller protein complexes have also been conducted using FRET microscopy. Some examples of such studies include the study of interactions of DNA/chromatin regulatory proteins, including the interaction of PCNA protein with SAS-I complex and Pol30 proteins [82], and crosstalk between Gal4 transcription factor and SAGA complex [83]. Most importantly, FRET biosensors have been used in understanding the assembly of proteins in the mitochondrion as well as vacuolar membranes [84,85]. The study of specific protein assemblies, such as the assembly of the lysosomal v-ATPase complex under predefined conditions, could lead to the discovery of novel mechanisms involved in impairing v-ATPase activity or help discover drugs that can modify the protein assembly. An example of such disruption of v-ATPase assembly in lysosomes can be observed in neurons of Alzheimer’s disease patients [45]. FRET-based biosensors may provide a suitable platform to identify drugs and compounds that can improve the assembly and disassembly of protein complexes involved in disease pathogenesis.

#### 3.2.4. Yeast G-Protein Coupled Receptor (GPCR)-Based Biosensors

In higher eukaryotes, GPCRs provide the ability to sense extracellular biomolecules involved in various processes, including peptides, hormones, small molecules, neurotransmitters, or even light [86]. Nearly 950 genes encoding human GPCRs have been identified, with a majority still to be explored for their functions [87]. The fundamental mechanism of GPCR signaling involves a seven transmembrane protein with seven alpha helices present in the transmembrane region and three loops in both the extracellular as well as intracellular space connecting these transmembrane domains [88]. The protein receptor is intracellularly coupled with heterotrimeric G-proteins. Once the ligand or the interacting molecule is recognized or sensed by the GPCR transmembrane protein, the heterotrimeric G-protein, consisting of Gα, Gβ, and Gγ subunits, undergoes conformational change resulting in the dissociation of Gα subunit from the complex [89]. The dissociated heterodimer of Gβ-Gγ then activates a mitogen-activated protein kinase (MAPK) cascade activating proteins downstream of the pathway and allows the expression of genes in response to the signal. In *Saccharomyces cerevisiae*, two such GPCR-mediated responsive mechanisms have been identified [90]. The first one activates in response to glucose (mediated by Gpr1p receptor) and the second one activates the pheromone response pathway (mediated by either Ste2p (α-factor receptor) or Ste3p (a-factor receptor) depending upon the mating type of the yeast strain used) [91]. The cascade of reactions following the GPCR mediated sensing and the way the GPCR activation triggers downstream cascades inside yeast cells are analogous to the GPCR signaling processes in higher eukaryotes, including humans [92]. Considering such conservation of the molecular pathway and the possibility of functional complementation of human GPCRs in yeast, the unicellular eukaryotes have been used for designing biosensors for various purposes.

Pioneering work in the development of yeast biosensors based on GPCRs has utilized the pheromone responsive pathway [93]. In yeast, pheromone factors are released as part of their mating behavior; these pheromone factors are recognized by the dedicated pheromone sensing GPCRs (Ste2p or Ste3p) on the cell surface [91]. The expression of human GPCRs instead of the yeast native GPCRs also activated the downstream pathways in the presence of native yeast Gα-proteins [94]; however, the efficiency of downstream signaling was increased by using chimeric Gα proteins, in which five residues of the native yeast Gα-protein at the C-terminus are replaced by the mammalian counterparts of the Gα protein corresponding to the GPCR being expressed [95]. The basic design of the GPCR-based biosensors includes the expression of mammalian GPCRs for sensing specific ligands/chemicals and a construct with a reporter gene under the control of a promoter that is activated by the GPCR signaling [43]. To avoid any unwanted consequences that could compromise the biosensor activity, the genes encoding the native GPCRs, proteins involved in the pheromone response pathway, and proteases that cleave GPCRs were deleted from the yeast GPCR biosensors [96]. In addition, some constructs were also inserted in a targeted manner to enhance the sensing ability of the biosensor. 

As an example, in a recent study, a yeast GPCR-based biosensor was designed to screen a compound library of agonists and antagonists that can target a human endocannabinoid receptor CB2 [96]. The researchers also analyzed a novel phytocannabinoid named dugesialactone and developed a portable device to detect drugs that can target the CB2 receptor. In the biosensor design, the GPCR receptor CB2 was expressed in a host strain where *STE3*, *SST2*, *FAR1*, and *GPA1* has been knocked out. Finally, the three-reporter system was designed for detecting the GPCR signaling via three different methods, including measurement of fluorescence, color, and luminescence. The reporter genes were controlled by the native pheromone responsive promoter from the *FIG1* gene. Apart from this example, more than fifty receptor-based biosensors have been designed in yeast, including the serotonin receptor-based biosensor, opioid receptor-based biosensor, and β2-adrenergic receptor-based biosensor [97,98,99].

The ease of growing yeast and engineering them to produce desired human GPCRs have an unprecedented impact on the production of GPCR-based biosensors for medical and biotechnological use. These biosensors, produced by expressing human GPCRs in engineered yeast, will allow simple, inexpensive, rapid, and high-throughput searches for novel therapeutics in coming years. 

## 4. Yeast Biosensors for Drug Discovery

The discovery of drugs is expensive as well as time-consuming. Drug discovery studies involve the identification of a drug target(s), validation, screening of drugs followed by safety and efficacy studies, testing drugs in animal models, and finally, conducting human clinical trials [100]. It has been estimated that just following the process and finding a drug will take around 10-15 years and cost hundreds of millions of dollars [100]. Yeast biosensors, in such instances, have proved to be inexpensive and safe systems. Biosensing yeasts in several studies involving the yeast two-hybrid system and yeast surface display have been successful in discovering drugs against neurodegenerative diseases, cancers, and several chronic conditions in humans [101,102]. As the two topics describing the yeast two-hybrid system and yeast surface display have already been described in previous sections, in this section, we attempt to describe systems that are different from the abovementioned examples. 

Yeast biosensors have been used as a suitable platform for screening drugs and compounds for diseases such as malaria and Alzheimer’s disease [103,104]. In a previous study targeting Alzheimer’s disease, statins have been tested for their ability to act against Aβ in yeast. Both the GFP tagged Aβ and native Aβ expressing yeast biosensors were tested against statins to compare the efficacy of these drugs in restoring the proteostasis balance after expression of Aβ in the yeast system [46]. The fusion protein, as well as the native protein contents in the cell, act as the biological part, and their turnover was evaluated to determine the effect of the drug treatment. 

Similarly, in other studies, yeast biosensors have been used to evaluate the effect of certain compounds such as tyramine and aluminum in yeast expressing Aβ on redox status [49,105]. The levels of reactive oxygen species inside the cells were evaluated using fluorescent dyes to determine the compounds’ effects. Mitochondrial health can also be monitored easily in yeast as they can survive with defective mitochondria; however, their ability to utilize carbon sources such as ethanol and glycerol that depend on mitochondrial respiration will be impaired [46]. Fluorescent dyes can also be used to visualize the mitochondria [106] and analyze cells for cellular defects [107]. In addition, yeast studies have also provided enormous information on fundamental systems such as autophagy that is conserved from yeasts to humans [45,108]. There are multiple methods based on fluorescent proteins and dyes available to monitor autophagy, which can aid in the discovery of drugs targeting diseases involving cellular proteostasis [46]. 

Yeast-based GPCR sensors can also be used in the future to screen compounds that can interfere with receptor function, as mentioned in the previous section. Compounds that can interfere with certain receptors, such as trace amine-associated receptor 1 in neurons, could be beneficial in identifying compounds that can modulate the dopamine and serotonin production levels in the synaptic cleft [109]. Such chemicals could be particularly beneficial in conditions such as Alzheimer’s disease, Parkinson’s disease, and schizophrenia, where these receptors are thought to have a substantial role in disease pathogenesis. 

## 5. Yeast Biosensors in the Environment and Biotech Industries

**Figure 8:**
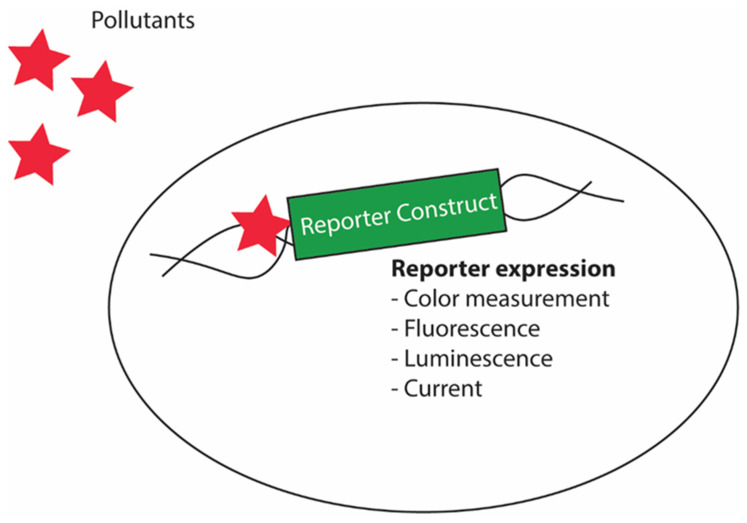
Schematic diagram showing yeast biosensor design for detecting pollutants. The system will sense the presence of the pollutants using pollutant sensing elements in the promoter of the biosensor design that enables expression of a downstream reporter gene.

The scheme of most biosensor designs is somewhat similar as they contain stimuli sensing elements and stimuli response elements. In the biosensor design, the stimuli sensing elements are the part such as promoters containing pollutant response elements, and the stimuli response elements refer to the reporter gene whose expression can be evaluated (Figure 8); however, some designs may be drastically different, but the overall underlying principle is similar. The pioneering application of yeast in environmental science involved its use in the measurement of biological oxygen demand (BOD) in wastewater [110,111]. BOD measurement in wastewater is a method to determine the levels of biodegradable organics in it. The standard method to detect the BOD takes around 5 days to give reliable measurement; yeast-based biosensors, in contrast, provide analogous reliable evaluation of BOD in minutes [44]. The basic principle of BOD measurement involves the measurement of the oxygen utilized during aerobic oxidation within a specified period [112]. Because yeasts can oxidize a broader range of organic compounds and considering their ability to withstand several toxic compounds, yeast biosensors are used to measure BOD. Hence, these sensors possess advantages over other microbial systems in measuring BOD in wastewater. The yeast biosensors allow the effective transfer of electrons to the electrode in the presence of mediator(s) such as ferricyanide [111]. This property of the yeast cells has been utilized to develop the yeast-based BOD sensors coupling with such mediators. The outputs of the biosensors are measured either by using oxidation reactions leading to chemiluminescence or by amperometry measurement [113]. Another similar application of yeasts was their use in biosensing the estrogenic activity of pollutants [112]. The estrogenic activity sensor was designed using a plasmid vector consisting of the reporter gene *lacZ*, which is expressed under the control of an inducible promoter with estrogen response elements and integrates the human estrogen receptor gene into the yeast genome. Once the pollutants encounter the yeast biosensor, the estrogenic activity of the pollutants activates the estrogen response elements, then activates the expression of a reporter gene. 

Furthermore, yeast biosensors are also used for the detection of metals in the environment using specific promoters such as the *CUP1* promoter, which activates readily in the presence of copper [114]. Reporter gene constructs controlled by *CUP1* promoter have been designed to study copper abundance using yeast biosensors. Apart from the abovementioned applications, yeast biosensors have also been applied to detect cadmium, marine toxins, and mycotoxins that pose threats to human health [115,116,117,118,119,120].

Several *Pichia pastoris* sensors have also been studied and designed for various purposes in biotech industries. Recently, *Pichia pastoris* has been used in identifying compounds that can interfere with the mosquito’s ability to sense the smell of its target [121]. In doing so, the *Pichia pastoris* genome has been engineered to express the mosquito’s olfactory receptor co-receptor (ORCO). Activation of the ORCO receptor by the stimulants causes an influx of calcium ion in the yeast sensors, which can be readily quantified by the fluorescent dyes that report on the calcium ions. The system can be used to identify compounds that inactivate the receptor as calcium influx will be decreased upon the interaction of the receptor with repellent chemicals. This is an epitome of how yeast can be applied to develop systems to explore rapid ways to identify compounds of human benefits.

A detailed list of biosensors and their applications in metabolic engineering have been reviewed elsewhere [122]. In this section, the attempt has been made to explain some of the unique yeast-based biosensors and provide insights into the design of such biosensors. Apart from the abovementioned examples, yeasts have been extensively utilized as cellular factories for synthesizing various compounds and biomolecules [1]. Motivated by conventional slow and tedious analytical techniques used to analyze product synthesis, determination of dynamic ranges, high producers, and phenotypic changes, the synthetic biotech industry required rapid and reliable alternatives. This urge has led to the discovery of yeast biosensors for use in synthetic biology along with other microbial sensors. Diverse types of yeast-based biosensors have been designed in the past. For example, a yeast GPCR-based biosensor was designed to study the microbial production of serotonin [123]. The mechanistic details of how a GPCR-based biosensor works have already been discussed in a previous section. Similarly, a TF-based yeast two-hybrid biosensor was created to sense the production of a class of compounds referred to as isoprenoids [124]. These compounds have no natural transcription factors making them difficult to assess for their evaluation in a biosensor; however, a methodology was developed incorporating the yeast two-hybrid system to activate a hybrid transcription factor that controls the expression of a reporter gene. In doing so, an enzyme, isopentyl diphosphate isomerase (IDI), that can bind to the ligands was identified. Fortunately, the enzyme dimerizes upon ligand binding, and this knowledge of how ligands dimerize the enzyme has been utilized to design the biosensor. In the biosensor, the IDI protein was fused with both the Gal4DBD and Gal4AD domains that are expressed separately, and a reporter was incorporated into the biosensor, which expresses a reporter gene under the control of the *GAL1* promoter. This biosensor allowed the detection of compounds that can interact with the IDI enzyme and cause its dimerization. This abovementioned example is a unique example of how a TF-based system has been combined with the yeast two-hybrid system to develop a novel strategy to screen compounds and ligands that target a particular protein of interest. 

## 6. Future Directions

The benefits of yeast being used as a biosensor have opened new avenues for drug discovery, understanding molecular pathways involved in disease pathogenesis, protein–protein interaction studies, understanding of the molecular architecture of complex protein assemblies, identifying mutations in proteins that have significance in determining the functional differences, and detecting pollutants from the environment. Yeast has already proved its benefits in studying protein–protein interactions, drug screening against several diseases, including cancer, Alzheimer’s disease, Parkinson’s disease, and others, detection of pollutants, and diagnosis of diseases. 

Recent advances in the use of these organisms in detecting infections of the SARS-CoV-2 virus are unprecedented. Yeast is the unique platform for studying compound/condition effects on mitochondrial health. Studies involving diseases with mitochondrial defects can be addressed in a better way using yeast sensors. Fluorescence reporters based upon fluorescent proteins have provided a basis for further development. For example, the concepts of novel FRET microscopy can be utilized further to develop rapid assays determining the protein proximity using the latest advancements in flow cytometers. In the future, it is expected that several multifunctional yeast biosensors can be easily developed using multiple designs inside a single yeast clone, which can be used for multiple investigations at a time. 

It is expected that a day will come when yeast can be used in the everyday diagnosis of diseases, not only human diseases but also those in domesticated animals. For instance, yeast can be designed in a way that it produces a reporter protein when dipped in blood or urine in the presence of disease-related molecules. 

Considering numerous prospects of using yeast as a biosensing tool, the yeast sensors will prove to be crucial in shaping our scientific advancements in the coming years. Although limitations such as differences in the post-translational modification machinery between the yeast and the source organisms for the protein of interest are required to be addressed, soon developments in the understanding of these processes will improve the biosensor’s sensitivity and specificity. Humanized yeast models producing proteins of interest with human post-translational glycosylation have already been produced and applied in the past [125]. In the future, it is expected that such improvements will continue to grow and will someday reach a point where answers derived from yeast will be identical to what is happening in humans reducing cost, labor, and time.

## Figures and Tables

**Table 1:** Some fluorescent proteins that have been used in yeast studies and their properties [62].

Fluorescent Protein	*Organism*	Molecular Weight (kDa)	Excitation Maxima (nm)	Emission Maxima (nm)	Brightness	pKa
**mTagBFP2**	*Entacmaea quadricolor*	26.7	399	454	32.38	2.7
**CFP**	*Aequorea victoria*	26.9	456	480	NA	NA
**Cerulean**	*Aequorea victoria*	26.8	433	475	26.66	4.7
**mTurquoise2**	*Aequorea victoria*	26.9	434	474	27.90	3.1
**mKeima**	*Montipora* sp. 20	25.1	440	620	3.46	6.5
**EGFP**	*Aequorea victoria*	26.9	488	507	33.54	6.0
**EYFP**	*Aequorea victoria*	27.0	513	527	44.89	6.9
**Venus**	*Aequorea victoria*	26.8	515	528	52.55	6.0
**mKO1**	*Verrillofungia concinna*	24.5	548	549	30.96	5.0
**tDimer2**	*Discosoma* spp.	52.7	552	579	81.60	4.8
**tdTomato**	*Discosoma* spp.	54.2	554	581	95.22	4.7
**DsRed/RFP**	*Discosoma* spp.	25.9	558	583	49.30	NA
**mRuby2**	*Entacmaea quadricolor*	26.5	559	600	42.94	4.4
**mStrawberry**	*Discosoma* spp.	26.6	574	596	26.10	4.5
**mCherry**	*Discosoma* spp.	26.7	587	610	15.84	4.5

NA in the table refers to “Not Available”.
